# Improvement in self-reported confidence in nurses’ professional skills in the emergency department

**DOI:** 10.1186/1757-7241-21-16

**Published:** 2013-03-05

**Authors:** Veli-Pekka Rautava, Erika Palomäki, Tapio Innamaa, Mika Perttu, Päivi Lehto, Ari Palomäki

**Affiliations:** 1 Department of Emergency Medicine, Kanta-Häme Central Hospital, Ahvenistontie 20, FI-13530, Hämeenlinna, Finland; 2 Faculty of Education, University of Jyväskylä, P.O. Box 35 (Educa/D), Jyväskylä, FI-40014, Finland; 3 Health Care Services of Hämeenlinna, Viipurintie 1-3, Hämeenlinna, FI-13100, Finland; 4 Department of Neurology, Kanta-Häme Central Hospital, Ahvenistontie 20, Hämeenlinna, FI-13530, Finland

## Abstract

**Background:**

The aim of this study was to assess nurses’ self-reported confidence in their professional skills before and after an extensive Emergency Department (ED) reform in Kanta-Häme Central Hospital.

**Methods:**

Emergency nurses participated in transitional training commencing two years before the establishment of the new organization in 2007. Training was followed by weekly practical educational sessions in the new ED. During this process nurses improved their transition skills, defined house rules for the new clinic and improved their knowledge of new technology and instruments. The main processes involving critically ill ED patients were described and modelled with an electronic flow chart software.

During the transitional training nurses compiled lists of practical skills and measures needed in the ED. These were updated after feedback from physicians in primary and secondary care and head physicians in Kanta-Häme Central Hospital. The final 189-item list comprised 15 different categories, each containing from 4 to 35 items. Based on the work described above, a questionnaire was developed to reflect ED nurses’ skills in clinical measures but also to estimate the need for professional education and practical training. Nurses working in the ED were asked to fill the questionnaire in January 2007 (response rate 97%) and in January 2011 (response rate 98%).

**Results:**

Nurses’ self-reported confidence in their professional skills improved significally in eight classes out of fifteen. These classes were cannulations, urinary catheterizations, patient monitoring, cardiac patients, equipment, triage and nurse practising, psychiatric patients as well as infection risk. Best results were noted in urinary catheterizations, patient monitoring and infection risk. When studying the group of nurses participating in both surveys in 2007 and 2011, improvements were observed in all fifteen categories. All but two of these changes were significant (p<0.05).

**Conclusions:**

During an extensive reform of emergency services, we noted a significant improvement in the professional skills of nurses. This improvement was especially consistent among nurses working in the ED during the whole transition process. Nurses’ education and training program in the ED may be successfully put into practice when based on co-operation between nurses and physicians dedicated to emergency services.

## Background

A substantial reform of emergency care took place in the province of Kanta-Häme in Southern Finland to ensure effective and immediate treatment of critically ill emergency patients. Three separate out-of-hours services in primary health care (PHC) and an old emergency department (ED) in the hospital were combined into one large ED in April 2007. The catchment population of the ED is 93000 inhabitants for primary care and 175000 for secondary care.

Several studies have shown the importance of accurate and immediate diagnosis and treatment of critically ill emergency patients [1-4]. An effective and well functioning pathway of care is essential e.g. for STEMI, stroke, severe trauma and sepsis patients [1,2,5-7]. Many studies have focused on the treatment of specific disorders [1-4,7]. There are, however, only a few studies evaluating the professional skills of nurses working in EDs.

The aim of this study was to assess nurses’ self-reported confidence in their professional skills before and after the ED reform.

## Methods

### Education and training

Transitional training commenced in 2005, i.e. two years before the establishment of the new organization. Participants were nurses, physicians, emergency trauma technicians and secretaries from the out-of-hours services in PHC and the ED in secondary care. In joint sessions skills for transition were improved and house rules for the new clinic were defined. Every nurse in the coming ED also visited beforehand both the ED in the hospital and the out-of-hours services in PHC. These visits involved following clinical work, becoming acquainted with the technology and instruments as well as holding discussions of working methods with nursing colleagues. Additionally, participants were encouraged to take part in a work exchange program, where nurses from primary care went for two to three weeks to work in the emergency room of the hospital and vice versa.

Nineteen of the most important processes involving critically ill ED patients were described and modelled with an electronic flow chart software (QPR Software^®^). This revealed potential problems and weaknesses in some pathways of care, this giving indications regarding the need for further training. Transitional training lasted from 2005 to 2007, and was followed by weekly practical educational sessions in the new ED. A group of nurses also participated in national congresses and specializing courses.

During the transitional training sessions nurses compiled lists of practical skills and measures needed in the ED. Further, a comprehensive list of professional skills was completed as described below. A systematic and regular education and training program was established on the basis of this output. Education and training program contained lectures (topics like principals of triage, interpreting ECG, treatment of trauma, cardiac and infection patients, eye procedures, burn wounds) and training (e.g. CPR, use of CPAP, BiPAP, infusion pump, assisting the doctor in inserting arterial line, chest tube and lumbar puncture). Education sessions and lectures were organized once a week and repeated to ensure every nurse had the possibility to participate in.

### Definition of practical skills and questionnaire

Local physicians, health care administrators and policy-makers defined the principles of the new ED. At the beginning of the transitional training, nurses discussed the various skills needed in the coming ED. They then participated in workshops where each group had its own specific topic (for example cardiac patients, ENT procedures, infection patients and trauma patients). Thereafter, they together further analysed which clinical measures nurses should be capable of managing in different situations. By such repetitive processing of topics we sought to ensure a wide coverage of different skills and measures. After this preliminary innovative work, a small group of nurses classified and sorted the topics with the guidance of the head physician of the ED (AP).

Physicians in both primary care and the hospital ED as well as head physicians (e.g. in anesthesiology, gynecology, internal medicine, neurology, pediatrics, psychiatry, pulmonology, orthopedics, and surgery) in Kanta-Häme Central Hospital were consulted. According to the feedback received, the lists of measures and practical skills were updated. The final output comprised 15 different categories of clinical measures and practical skills, each containing from 4 to 35 items (Table 1). There were altogether 189 different measures in these categories (Additional file 1).

**Table 1:** Classification of clinical measures and professional skills in emergency department (in brackets the number of different measures in each category)


**A. **Cannulations (14)	**F. **ENT procedures (10)	**K. **Triage & nurse practicing (22)
**B. **Punctures (9)	**G. **Eye procedures (6)	**L. **Critical patients (18)
**C. **Urinary catheterization (4)	**H. **Patient observation (9)	**M. **Psychiatric patients (8)
**D. **Gastrointestinal tubes (6)	**I. **Cardiac patients (20)	**N. **Infection risk (5)
**E. **Recognition of instruments (5)	**J. **Equipment (18)	**O. **Immobilization (35)

Based on the work described above, a questionnaire was developed to reflect ED nurses’ skills in clinical measures but also to estimate the need for professional education and practical training. Respondents were asked to assess for each of the 189-items whether they:

A. can independently carry out the specific measure without the help of a more experienced ED nurse

B. can undertake the specific measure with the advice or help of a more experienced ED nurse

C. cannot manage the specific measure.

Each individual nurse had to fill in the questionnaire, which was given to them by the head nurse of the clinic. Everyone had enough time during the working day to fill in the questionnaire. We wanted to ensure the truthfulness of the answers. The staff was informed before filling in the questionnaire, that option A might effect on nurses’ work and increase their responsibilities during the shift. Further it was made clear that option C was not a sign of possible personal incapability but an indicator to the employer to arrange adequate in-service training for the personnel.

The aim of this study was to assess nurses’ self-reported confidence in their professional skills before and after the ED reform. Particularly we sought to establish whether there was

1) A change in self-reported confidence in professional skills among nursing staff in the ED in 2007 and 2011,

2) A change in self-reported confidence in professional skills within a group of nurses participating in both questionnaires in 2007 and 2011 (“experienced” group),

3) a difference between a group of nurses participating in both questionnaires (“experienced” group) and a group participating in only 2011 (“new” group).

### Participants

Participants in this study were nurses working in the ED in 2007 or 2011. The questionnaire was first filled in January 2007 and repeated in January 2011. Thirty-six nurses from the ED filled in the first questionnaire and 58 the second. A total of 22 nurses worked in the ED both 2007 and 2011 and participated in both surveys. Details of the participants are shown in Table 2.

**Table 2:** Nurses participating in the study

**Variable**	**Year**		**Subgroups of Nurses in 2011**	
	2007	2011	“New”	“Experienced”
Nurses	36	58	36	22
Women/Men	32/4	46/12	26/10	18/4
Age, years	39.2 ± 9.3	38.6 ± 10.5	35.5 ± 10.2	43.7 ± 9.1**
Professional experience, years	11.3 ± 8.8	10.6 ± 8.9	7.7 ± 7.9	15.4 ± 8.5***

Continuous variables are presented as mean ± SD.

P values between “new” and “experienced” nurses were as follows: ** p < 0.01; *** p < 0.001.

### The processing of the answers and statistical methods

Every questionnaire with answers was collected, processed and coded by the clinical secretary working in the hospital but not in the ED. In the analysis the different answer options gave points as follows: option A gave two points, option B one point and option C no points. Points of the answers were imported to the spreadsheet software (Microsoft Office Excel^®^). The individual points in each class were summed and divided with theoretical maximum points of the class. Because categories had different amount of practical skills and measurements, the results were finally presented as percents. The maximum result was thus 100 in every category.

Statistics were analyzed with SPSS Statistics 20 (IBM 2011). Results are presented as mean ± standard error of mean (SEM) if not stated otherwise. A non-parametric Mann Whitney U-test was used in assessing differences between results from 2007 and 2011. Wilcoxon signed rank test was used in paired comparisons. Statistical significance was accepted at p value < 0.05.

## Results

**Figure 1:**
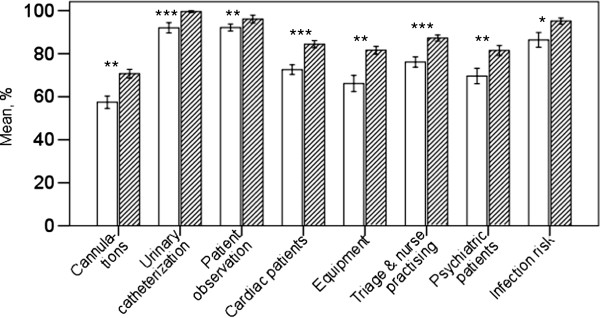
**Presentation of the eight practical skills with significant improvement from 2007 to 2011.** Maximum result in each skill is 100%. *** p<0.001, ** p<0.01, * p<0.05. Non-shaded bars: 2007, shaded bars: 2011.

Response rate was 97% (36/37) in 2007 and 98% (58/59) in 2011. Nurses’ self-reported confidence in their professional skills improved significally in eight classes out of fifteen (Figure 1). The best results were noted in urinary catheterization, patient observation and charting as well as infection risk, figures being more than 90 per cent in the second evaluation (year 2011). The theoretical maximum was 100% in each category meaning in practice that every nurse had chosen option A in every skill or measure included in the category.

**Figure 2:**
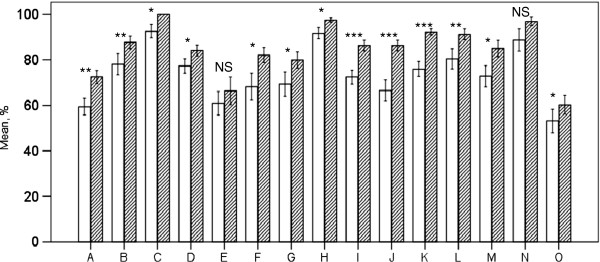
**Change in skills in those nurses practising in the clinic in both 2007 and 2011. **Letters in x-axis for classification of clinical measures and professional skills are presented in Table 1. Maximum result in each skill is 100%. *** p<0.001, ** p<0.01, * p<0.05, NS: non-significant. Non-shaded bars: 2007, shaded bars: 2011.

In the group of nurses participating in both inquiries we observed improvements in all fifteen classes. All but two of the changes in the skills of “experienced” nurses were significant (p<0.05). The positive change in recognition of instruments and infection risk remained non-significant (Figure 2). When comparing the groups of “experienced” and “new” nurses we noted a significant difference in five categories of skills. “Experienced” nurses were significantly more self-confident in the classes of punctures, eye procedures and triage (all p values <0.01) as well as in the ENT procedures and equipments (both p values <0.05).

## Discussion

The main finding in this study was a significant improvement in nurses’ self-reported confidence in their professional skills from 2007 to 2011. This corresponds well with the findings in previous studies in health care, showing the positive effect of systematic and scrutinized education. Groups under Källestedt and Preusch reported an increase in resuscitation skills among health care professionals after training [8,9], and Lam and associates an improvement in doctors’ confidence to diagnose and manage common dermatology problems after a Certificate Course in Clinical Dermatology designed for primary care doctors [10]. In our study in question the skills were divided into 15 categories. Improvements were revealed in 13 categories, of which eight were significant.

It was encouraging to note here an improvement in professional skills in widely different areas. There was notable progress with cannulations (+22%), cardiac patients (+15%), equipment (+22%), psychiatric patients (+14%) and infection risk (+11%). These improvements can be attributed to systematic and scrutinized training. For example in cardiac care there was abundant training because patients with acute cardiac problems are very common in the clinic. Since these patients are treated by all nurses, personnel gain extensive experience in this area. On the other hand, even though we found evident improvement in practical skills in cannulations, work evidently remained to be done, since the overall confidence in this area was only 70 per cent. This can be explained in terms of the number of operations, involved, for example preparing the patient for kidney transplantation, of which are very few in our clinic.

The rareness of certain operations in the emergency clinic would also appear to explain lack of confidence in some other categories. Minor ear, nose and throat (ENT) and eye operations in our hospital are usually performed in the outpatient clinic for elective patients, while in the emergency clinic they do not occur every day. These operations are also assigned to nurses who have more experience of them. By reason of the unusualness of more demanding ENT and eye operations within these categories the overall confidence remained low. Confidence was lowest in traumatology and in the recognition of instruments. The category involving measures in the field of the immobilization of trauma patients was fairly wide and included tasks which in Finland are undertaken by senior emergency trauma technicians. Hence the nurses participating in this study would not usually carry out those operations in our clinic. Among all participants, recognition of instruments was the only category where the confidence had an evident, albeit non-significant tendency to decrease. Our finding is in line with those in a study carried out among physicians working in emergency medical service [11]. It can be explained by the considerable turnover in the personnel in our emergency clinic and thereby the lack of practical experience.

Confidence was particularly high (almost 100 per cent) in the fields of urinary catheterization, observation of the patient and infection risk. Patient observation was the only category, in which “new” nurses seemed to feel non-significantly more confident than more experienced staff. Nurses in “new” group had less practical experience. It might in some cases lead to the self-deception described by Baxter and Norman, who found a lack of association between self-assessment and actual performance among nursing students [12]. In 2007 the mean number of years after graduation was 11.3, and in 2011 it was 10.5. Although the mean difference was not conspicuous, it is noteworthy that in 2011 there were a considerable number nurses just graduated. This can be seen in the medians of experience in 2007 and 2011, 8.85 and 7.35 years, respectively.

Nurses who worked in both 2007 and 2011 felt their professional confidence increased in all categories, in 13 of them significantly. These improvements also included significant changes in six categories which were not significant among the nurses of the whole clinic. In infection risk the confidence was in 2011 almost 100%, but the change did not reach statistical significance.

As there was a notable turnover in personnel we also compared the confidence of the nurses who worked in both 2007 and 2011 to those who only worked in 2011. This revealed that experienced nurses had a tendency to be more confident in all categories but one, although the difference was significant in only five categories. These categories were punctures, ENT and eye procedures as well as equipment and triage. Experience seemed to have an influence on nurses’ confidence. This observation is supported by earlier findings by George and colleagues, who studied doctors’ confidence levels in diagnosing diabetes [13]. They reported that those doctors who had had post graduate training in diabetes obtained a higher score in confidence.

The basic idea was to assess nurses’ self-reported confidence in their professional skills as a matter of the organization, not of the individual nurses. Some of the nurses in the “new” group had experience of years in working in our ED or in other EDs. It could be possible, that non-confident nurses had left the ED in order to work elsewhere and were replaced by more confident nurses. However, during the study period many experienced nurses reached their retiring age and were obliged to leave the department. Therefore, the turnover in personnel caused a potential risk of decreasing competence of the staff.

The strength of this study is that the questionnaire was devised by collaboration among multiple professionals. Nurses, physicians, emergency trauma technicians and ED secretaries all participated in the preparation of the questions. In this way we were able to cover as many skills as possible. Since the individual questions were explicit, the possibility of misunderstandings was minimal.

The focus of this study is quite new in the field of emergency medicine, and we had no established and validated questionnaire. The confidence scale we used involved only three choices, which made it easy and quick to answer. We also sought to avoid e.g. a Likert-type scale, as this would have made it difficult to assess results. Using a scale differing from those used in schools prevented nurses from answering according to their previous grades.

In the questionnaire nurses’ clinical responsibility was emphasized and related to the confidence scale. When nurses assessed their confidence in handling certain operations they also had to consider whether they could be the nurse in charge in that operation during the shift.

We were concerned in this inquiry to avoid feelings of guilt or shame if a participant could not carry out certain measure or lacked some practical skill. Answer C “cannot manage the specific measure” was related to the responsibility of employer to provide education and practical training. We believe this written principle contributed to the truthfulness of the answers.

To our knowledge there have been no previous studies dealing with improvements in the confidence of nurses in one whole ED. We examined the confidence of all the nurses working in the department and were able to establish the overall level of confidence in our clinic.

The use of self-reported confidence instead of an objective measure is a limitation to this study. Previous studies have brought out concerns regarding evaluating professional skills and competence by self-assessment. Some studies carried out among medical and nursing students have questioned the correlation between self-assessments and actual performances [12,14]. Davis and associates have compared 17 studies to determine how accurate physicians’ self-assessments are compared to external observations of their competence [15]. Thirteen comparisons out of 20 (three studies used two external comparisons) showed little or no connection between self-assessments and external observations. In seven studies a positive association was noted. To avoid such previously noted shortcomings, we tied the questions to practical responsibility by asking whether respondents felt confident to be the nurse in charge during each operation.

Meretoja and Koponen have made a study designed to develop a model to compare nurses’ optimal and actual competence [16]. A multiprofessional expert group defined what was the optimal competence needed in a perioperative care setting. This was compared to nurses’ and nursing managers’ assessments of nurses’ actual competence. A significant difference was found between nurses’ optimal and actual competence. Also nurses’ self-assessed competence was lower than their competence as assessed by nursing managers.

The goals for learning were defined before the clinical reform of the ED in spring 2007. We were thus obliged to guess what skills nurses would need in the new department. The items included in the questionnaire might therefore not fully correspond to the skills needed in real life. However, according to our experience they covered surprisingly well the skills and measures needed in the ED today. Since the reform we have encountered new machines, knowledge and experience which would help us to devise more extensive questions. In the present questionnaire there were also some operations which for nurses are good to know about but which come up only rarely. For example assisting in the insertion of a pacemaker or emergency cricothyroidotomy are not common in the clinic. Such operations have probably lowered the overall confidence.

Our questionnaire comprised 15 different categories. The number of questions per category varied from 4 to 35. This might have had an influence on the statistical significance of the findings in different categories. Nevertheless, such influence would not appear to have been particularly marked, since we found significant improvements in categories with both few and numerous questions.

Even though the improvements in nurses’ professional skills might not be as good as they assume, we believe they are real. Such a conception is supported by the fact that the improvements were extensive and systematic. There was improvement especially in areas the nurses working frequently.

## Conclusions

In this study, taking place during an extensive reform of emergency services, we found a significant improvement in the professional skills of nurses working in the ED. This improvement was especially uniform among nurses working in the ED during the whole transition process.

According to our results, nurses’ education and the training program in the ED may be successfully put into practice when based on the co-operation of nurses and physicians dedicated to emergency services.

## Abbreviations

BiPAP: Bilevel positive airway pressure; CPAP: Continuous positive airway pressure; CPR: Cardiopulmonary resuscitation; ECG: Electrocardiography; ED: Emergency Department; ENT: Ear, nose and throat; PHC: Primary health care; SEM: Standard error of mean; SD: Standard deviation; STEMI: ST elevation myocardial infarction.

## Competing interests

The authors have no competing interests.

## Authors’ contributions

AP designed the study. PL and AP participated in the acquisition of data. VP-R, MP, EP and AP participated in the analysis of the data. VP-R, EP, TI and AP drafted the manuscript. All authors have read and approved the final manuscript.

## Supplementary Material

Additional file 1 Logbook for nursing staff or goal for learning. Click here for file
