# Supplementary material for: Improvement in self-reported confidence in nurses’ professional skills in the emergency department
Source: Scand J Trauma Resusc Emerg Med. 2013 Mar 5;21:16. doi: 10.1186/1757-7241-21-16 (PMC3599318; doi:10.1186/1757-7241-21-16)
Supplement: Additional file 1 — Logbook for nursing staff or goal for learning. [file 1757-7241-21-16-S1.doc]

**Logbook for nursing staff or goal for learning**

1. **Cannulations**
2. Peripheral venous access
   1. Inserting IV line
   2. IV infusions fluids / medicines kvo (keep vein open)
   3. Fluid/medicine infusion with calculator
3. Arterial cannulas
   1. Inserting arterial line, assisting
   2. Measuring invasive arterial pressure
4. Central IV line
   1. Inserting central IV line, assisting
   2. Measuring and following the CVP
5. Implanted venous access port
   1. Infusion management
   2. Drawing blood sample
   3. Heparin lock flush -injection
6. Dialysis
   1. Turbid peritoneal dialysis fluid; recognition and procedures
   2. Assisting the patient or the relative with peritoneal dialysis
   3. Procedures with tunneled CV dialysis catheter
   4. Preparing the patient for kidney transplantation
7. **Punctures (including assisting when taking samples: collecting the right tubes and basins, lab requests)**
8. Pleurocentesis
   1. Assisting pleurocentesis, forwarding the samples
   2. Inserting chest tube
9. Sternal puncture; preparing the patient and assisting in the procedure
10. Lumbar puncture; preparing the patient and assisting in the procedure
11. Maxillary puncture; preparing the patient and assisting in the procedure
12. Intra articular injections (including sampling) ; preparing the patient and assisting in the procedure
13. Paracentesis for ascites aspiration; preparing the patient and assisting in the procedure
14. Cystocentesis
    1. Preparing the patients, assisting in the procedure and sample taking
    2. Changing suprapubic bladder drainage (Cystofix®); assisting the doctor
15. **Urinary catheterization**
16. In and out catheterization
17. Inserting permanent urinary catheter
18. Urinary bladder irrigation
    1. Inserting the irrigation catheter
    2. Irrigating the bladder
19. **Gastrointestinal tubes**
20. Nasogastric (N-G) tube
    1. Inserting N-G tube for adult
    2. Inserting N-G tube for child
    3. Performing gastric lavage and observing the patient
21. Changing percutaneus endoscopic gastrostomy (PEG) tube
22. Rectal tube
    1. Inserting rectal tube
    2. Enema
23. **Recognition of instruments (pictures, colloquial names)**
24. GYN instruments
25. Surgical instruments
26. Ear, nose and throat (ENT) instruments
27. Ophthalmologic instruments
28. Anesthetic instruments
29. **ENT procedures**
30. 1 Epistaxis
    1. Treatment of epistaxis; first aid
    2. Silver nitrate coagulation; instruments, supplies and assisting
    3. Anterior tamponation; instruments, supplies and assisting
    4. Posterior tamponation; instruments, supplies and assisting
    5. Observation of epistaxis patients, BP, Hb
31. Peritonsillar abscess, tonsillar bleeding
    1. Treatment of peritonsillar abscess; assisting in the procedure and taking samples
    2. Follow-up of peritonsillar abscess patient
    3. Recognition of postoperative tonsillar bleeding
    4. Treatment of tonsillar bleeding with diathermia; assisting in the procedure
32. Rapid ultrasonic diagnosis of maxillary sinusitis (use of Sinuscan™)
33. **EYE Procedures**
34. Immediate ocular flushing
    1. Indications for ocular flushing and recognition of immediate need for treatment
    2. Performing the procedure
35. Removal of foreign body from the eye
    1. Turning the eyelid inside out and removing a FB
    2. Using the FB needle or drill; assisting
36. Assisting in microscopic eye examination
37. Recognition of the common eye medicines
38. **Observation of the patient and charting**
39. Observation of the unconscious patient, Glasgow Coma Scale (GCS)
40. Estimation and follow-up of pain, visual analogic scale (VAS)
41. Follow-up of respiration
    1. Oxygenation (SpO2-pletysmography)
    2. Ventilation (EtCO2- capnography)
42. Follow-up of circulation
    1. Blood pressure
    2. Heart rate (dysrythmias, ischemia of heart)
    3. Temperature of the peripheral limbs
    4. Diuresis
43. Follow-up of blood glucose, taking temperature regularly
44. **Cardiac patient, ECG machines and pacemakers; operation and interpretation**
45. Taking ECG
    1. Acquisition 12 lead ECG, recognizing reasons for errors
    2. Taking special leads, V4R
    3. Taking pacemaker patient ECG, using magnet
46. Interpreting ECG
    1. Ventricular fibrillation
    2. Ventricular tachycardia
    3. Atrial fibrillation and flutter
    4. Supraventricular tachycardia
    5. Complete AV block
    6. STEMI - ST Segment Elevation Myocardial Infarction
    7. ST depression, possibly due to ischemic
47. Monitoring ECG
    1. Bed-side monitoring
    2. Use of central monitoring unit
48. Operating defibrillator
    1. Manual defibrillation
    2. Semi-automatic defibrillator
49. Using temporary internal pacing
    1. Recognizing the instruments and preparation for procedure
    2. Assisting in the insertion of the pacemaker
    3. Follow-up of the pacemaker patient
50. Use of temporary external pacemaker
51. Cardioversion; assisting
    1. Pharmacologic cardioversion (medicines and doses)
    2. Electrical cardioversion (IV-anesthesia, assisting in the anesthesia and procedure)
52. **Other Equipment, Preparation, Operation and Assisting**
53. Ultrasound (US) examinations
    1. Measuring residual urine
    2. Preparation for lower extremity Doppler examination
    3. Preparation for echocardiography
    4. Gynecologic US examination and assisting in sample taking (PAPA, Pipelle®)
    5. Preparation for abdominal US
    6. Preparation for pleural US
54. Diathermy
    1. Surgical diathermy; preparation and assisting
    2. ENT diathermy; preparation (special tip) and assisting
55. IV therapies
    1. Use of infusion pump
    2. Use of syringe infusion pump
56. Use of blood warmer
57. CPAP machine; preparation and treatment
    1. CPAP with mask
    2. CPAP for intubated patient
58. Use of ventilator (Oxylog®)
    1. Attaching the tubes and testing
    2. Basic setting and start
    3. Performing CPAP treatment
    4. Performing BIPAP treatment with mask
    5. Performing BiPAP treatment for intubated patient
59. **Triage and duties of a nurse for general practice patient**
60. Principles of triage (category A-E, duties of a nurse)
61. Carrying out triage in practice
62. Principles for sick leave and transportation document
63. Upper respiratory tract infection
    1. Flu, bronchitis etc
    2. Use of Sinusscan® (see F3.)
64. Urine track infection
65. Bacterial infections of the skin
66. Using pain medication
    1. Non-steroidal anti-inflammatory drugs
    2. Centrally acting analgesic and narcotics
67. Fluid therapy for febrile patient
    1. Pediatric gastroenteritis
    2. Other pediatric febrile diseases
    3. Rules for adult fluid therapy
68. Measurements helping the nurse
    1. Quick blood glucose test
    2. Quick-CRP
    3. Quick Hb test
    4. Alcometer
    5. PEF
    6. Quick test for throat streptococcus infection
69. Recognizing immuno-compromised patient
70. Estimating the need for child protection report
71. Institutions and treatment facilities for GP patients and senior citizens
72. Pharmacologic course and examination at Central Hospital of Kanta-Hame

(2=participated and passed, 1= participated, 0=not participated)

1. **Critical or Seriously Sick Patient**
2. Cardio-pulmonary resuscitation (CPR)
   1. Recognition of cardiac arrest
   2. Ventilation-chest compression resuscitation, adult and child
   3. Medicines and devices in CPR
3. Intubation
   1. Recognition of intubation tubes, stylets and tongue forceps
   2. Use of suction
   3. Assisting doctor in intubation and ventilation
4. Emergency cricothyroidotomy
   1. Recognition of the instruments
   2. Assisting the doctor in the procedure
5. Hemorrhagic patient; immediate treatment, investigation and follow-up.
6. Unconscious patient; immediate treatment, investigation and follow-up.
7. Chest pain patient; immediate treatment, investigation and follow-up.
8. Dyspneic patient; Immediate treatment, investigation and follow-up.
9. Treatment sequence of stroke patient
10. Immediate treatment and follow-up of ketoacidotic patient
11. Treatment and follow-up of poisoned patient
12. Treatment and follow-up of combustion patient
13. Trauma team of Central Hospital of Kanta-Häme (treatment sequence of trauma patient)
14. Catastrophe plan; action expected according to my profession
15. **Psychiatric and intoxicated patients**
16. Ability to support and comfort the patient and relatives in critical situation
17. Intoxicated patient
    1. Recognizing intoxicated patient
    2. Treatment for acute intoxicated patient in outpatient clinic
    3. Mini-intervention for intoxicated patient
18. Treatment institutions
    1. Knowing the GP clinic, clinic for intoxicated patient and social welfare offices; guiding patients to visit them.
    2. Awareness to utilize social service on call
19. Capability to recognize psychotic patient
20. Ability to follow-up mentally sick patient in out-patient clinic
21. **Infection Risk**
22. Principles of hand hygiene
23. Isolation of contagious patient
24. Isolation in practice in emergency department
    1. Contacts with patients
    2. Taking samples
    3. Taking care of nourishment
25. **Immobilization**
26. Trauma patient
    1. Transferring the patient from the ambulance stretcher
    2. Use of trauma stretcher
    3. Use of spinal board
    4. Use of lumbar belt
27. Splinting
    1. Wrist splint
    2. Finger splint
    3. Knee support
    4. Patella support
    5. Ankle support
    6. Pad bandage
    7. Back slab
28. Supportive bandages
    1. Adhesive elastic bandage for ankle
    2. Collar and cuff
29. Neck support
    1. Neck support for bed patient
    2. Stiff neck support
    3. Soft neck support
30. Traction
    1. Assembling tibia traction
    2. Preparation and follow-up of tibia traction patient
    3. Assisting in tibia traction
    4. Assembling skull traction devices
    5. Preparation of skull traction patient and follow-up
    6. Assisting in the skull traction procedure
31. Restraint devices
32. Plaster and reduction
    1. Short plaster boot for lower extremity
    2. Long plaster boot for lower extremity
    3. Plaster cylinder for lower extremity
    4. Fiberglass and plaster of Paris (POP)
    5. Angle plaster for upper extremity
    6. U-plaster for upper extremity
    7. Orthosis of upper arm
    8. Radial splint
    9. POP for scaphoideus fracture
33. Dislocations
    1. Treatment of hip dislocation
    2. Treatment of humerus dislocation
    3. Treatment of finger and toe dislocations
